# Usefulness of procalcitonin at admission as a risk-stratifying biomarker for 50-day in-hospital mortality among patients with community-acquired bloodstream infection: an observational cohort study

**DOI:** 10.1186/s40364-023-00450-3

**Published:** 2023-01-17

**Authors:** Abderrahim Oussalah, Jonas Callet, Anne-Elisabeth Manteaux, Nathalie Thilly, Nicolas Jay, Jean-Louis Guéant, Alain Lozniewski

**Affiliations:** 1 Department of Molecular Medicine, Division of Biochemistry, Molecular Biology, and Nutrition, University Hospital of Nancy, Rue du Morvan, F-54511 Vandoeuvre-lès-Nancy, France; 2 Nutrition, Genetics, and Environmental Risk Exposure (NGERE, INSERM UMR_S 1256), Faculty of Medicine of Nancy, University of Lorraine, INSERM, 9, Avenue de la Forêt de Haye, F-54511 Vandoeuvre-lès-Nancy, France; 3 Department of Microbiology, University Hospital of Nancy, Rue du Morvan, F-54511 Vandoeuvre-lès-Nancy, France; 4 Department of Methodology, Promotion and Investigation, University Hospital of Nancy, Rue du Morvan, F-54511 Vandoeuvre-lès-Nancy, France; 5 Department of Medical Informatics, University Hospital of Nancy, Rue du Morvan, F-54000 Vandoeuvre-lès-Nancy, France; 6 Orpailleur, LORIA UMR 7503, Vandoeuvre-lès-Nancy, F-54000 Nancy, France; 7 Stress Immunity Pathogens Laboratory (EA7300), Faculty of Medicine of Nancy, University of Lorraine, 9, Avenue de la Forêt de Haye, F-54511 Vandoeuvre-lès-Nancy, France

**Keywords:** Procalcitonin, Risk-stratifying biomarker, 50-day in-hospital mortality, Community-acquired bloodstream infections

## Abstract

**Purpose:**

To assess the association between plasma procalcitonin concentration at hospital admission and the risk of 50-day in-hospital mortality among patients with community-acquired bloodstream infections.

**Methods:**

We carried out a retrospective, observational cohort study with all consecutive patients with bacteriologically confirmed community-acquired bloodstream infections hospitalized between 2006 and 2012. We aimed to assess the association between plasma procalcitonin at admission and 50-day in-hospital mortality. Patients were included in the analysis if they had undergone a blood culture test within 48 hours of hospitalization with a concomitant procalcitonin assay (time < 12 hours between the two tests). Inclusion in the study began on the day of hospital admission, and each patient was followed until death, discharge from the hospital, or last known follow-up in the 50 days following hospital admission. The endpoint was the occurrence of all-cause in-hospital mortality during the 50 days following hospital admission.

**Results:**

During the 7-year study period, 1593 patients were admitted to one of the healthcare facilities of the University Hospital of Nancy from home or through the emergency department and had positive blood cultures and concomitant procalcitonin assays. Among the patients, 452 met the selection criteria and were analyzed. In ROC analysis, procalcitonin at baseline was significantly associated with 50-day in-hospital mortality, with an optimal threshold > 4.24 ng/mL. A baseline procalcitonin > 4.24 ng/mL was independently associated with an increased risk of in-hospital mortality (multivariable logistic regression: odds ratio, 2.58; 95% CI, 1.57–4.25; *P* = 0.0002; Cox proportional hazard regression: hazard ratio, 2.01; 95% CI, 1.30–3.11; *P* = 0.002). In sensitivity analyses, baseline procalcitonin quartiles were independently associated with 50-day in-hospital mortality (multivariable logistic regression: odds ratio, 1.47; 95% CI, 1.17–1.85; *P* = 0.001; Cox proportional hazard regression: hazard ratio, 1.31; 95% CI, 1.07–1.60; *P* = 0.008). The independent associations between baseline procalcitonin and the risk of 50-day in-hospital mortality were maintained after adjusting for C-reactive protein and sepsis status at admission.

**Conclusion:**

Our data provide the first evidence of the usefulness of plasma procalcitonin at admission as a risk-stratifying biomarker for predicting 50-day in-hospital mortality among patients with community-acquired bloodstream infections.

**Supplementary Information:**

The online version contains supplementary material available at 10.1186/s40364-023-00450-3.

## Introduction

Sepsis is a leading cause of death worldwide, with more than 48 million incident cases and a mortality rate of up to 50% [1–4]. Community-acquired infections contribute to 70% of cases of sepsis and represent one of the leading causes of hospitalization in intensive care units [5, 6]. Bloodstream infections are diagnosed in a high proportion of patients with community-acquired sepsis and septic shock [7, 8]. Bloodstream infections represent a significant source of infectious disease morbidity and mortality worldwide, with an estimated overall incidence of 43 to 101 per 100,000 and a mortality rate of 15 to 30% [8–15]. A prospective, multicenter, observational study identified a bloodstream infection in 20% of patients admitted to intensive-care units with community-acquired sepsis [16]. The presence of bloodstream infections at admission was associated with an 86% increased risk of mortality in the ICU [16].

According to current guidelines, risk stratification and prognostication are paramount in patients with septic conditions, as high-risk patients may benefit from earlier clinical interventions [1, 6, 17, 18]. In a previous large cross-sectional study using the Nancy Biochemical Database [19–22], we analyzed more than 35,000 consecutive patients who underwent concomitant procalcitonin assays and blood cultures for suspected bloodstream infections. We demonstrated that plasma procalcitonin was useful for excluding bloodstream infections with negative predictive values ranging from 98.9 to 99.9%, according to pathogen categories [19].

To date, no study has assessed the usefulness of plasma procalcitonin at admission to predict in-hospital mortality among patients with community-acquired bloodstream infections (CA-BSIs). Using data from the Nancy Biochemical Database [19–22], we evaluated the usefulness of procalcitonin at admission as a risk-stratifying biomarker for predicting 50-day in-hospital mortality among patients with CA-BSIs.

## Methods

### Study population

All consecutive patients hospitalized in 67 healthcare departments of medicine, surgery, and obstetrics between January 1, 2006, and December 31, 2012, were considered for inclusion in the study. These patients were identified using the ‘Nancy Biochemical Database’, a prospectively maintained electronic database that collects the biochemical results of consecutive patients hospitalized in 67 healthcare departments of medicine, surgery, and obstetrics at the University Hospital of Nancy [19–22]. The “Nancy Biochemical Database” is registered at the French National Commission on Informatics and Liberty, CNIL, under record #1763197v0. The Ethics Committee of the University Hospital of Nancy approved the study.

### Study design, setting, and inclusion criteria

We carried out a retrospective, observational cohort study on all consecutive patients with a bacteriologically confirmed bloodstream infection hospitalized between 2006 and 2012. Inclusion in the study began on the day of hospital admission, and each patient was then followed until death, discharge from the hospital, or last known follow-up in the 50 days following hospital admission. The inclusion criteria were i) admission to one of the healthcare facilities of the University Hospital of Nancy between January 1, 2006, and December 31, 2012; ii) admission from home or through the emergency department; iii) positive blood cultures from blood drawn within 48 hours after hospital admission; and iv) concomitant blood culture and procalcitonin assay with an interval of less than 12 hours between the two tests. The exclusion criteria were i) nosocomial bloodstream infection diagnosed when one or more cultures of blood drawn at least 48 hours after admission yielded a pathogenic organism [10]; ii) blood culture contamination defined by the detection of microorganisms classically considered “potential contaminants found in blood cultures” according to the definition of Lee et al. [23]; and iii) no available follow-up data in the 50 days following hospital admission. The study was observational, i.e., all clinical assessments, biochemical explorations, and blood cultures were conducted at the discretion of the physicians of each healthcare department as part of a standard assessment for suspected bloodstream infection. The data collected and the methods used for procalcitonin assays and blood cultures are reported in the Additional file 1.

### Study aim and endpoint

The study aimed to assess the association between plasma procalcitonin concentration at hospital admission (considered baseline) and in-hospital mortality during the 50 days following hospital admission. The endpoint was the occurrence of all-cause in-hospital mortality during the 50 days following hospital admission.

### Statistical analysis

All quantitative variables are described as medians and percentiles [interquartile ranges (IQRs), 25–75th percentiles], and the qualitative variables are described as percentages and 95% confidence intervals (95% CIs). Comparing baseline plasma procalcitonin values between patients according to the occurrence of death was performed using the Wilcoxon Mann–Whitney test. We explored the optimal threshold of procalcitonin associated with the occurrence of death by performing a receiver operating characteristic (ROC) analysis, according to DeLong et al. [24], using the study endpoint as a classification variable. The optimal threshold was defined using the Youden index J. Bias-corrected and accelerated (BC_a_)-bootstrap intervals after 10,000 iterations were performed for the Youden index and its associated values [25].

In univariate analyses, we compared patient characteristics according to the presence or absence of 50-day in-hospital mortality (study endpoint) using the Wilcoxon Mann–Whitney test for continuous variables and the Chi-squared test or Fisher’s exact test, as appropriate, for categorical variables. All the significant variables in univariate analyses were integrated into a multivariable logistic regression model to identify independent predictors of 50-day in-hospital mortality using the forced entry or the stepwise method. We used age, pathogen genera identified in blood cultures, C-reactive protein, and sepsis status at admission as covariates for adjustment. All variables with *P* < 0.2 were included in the model and the variables with *P* < 0.05 were retained in the model. The results are shown as odds ratios (ORs) and 95% CIs for each predictor and the percentage of cases correctly classified by the logistic regression model. We assessed model discrimination using ROC analysis and the percentage of cases correctly classified by the model. We assessed the model’s goodness of fit using Nagelkerke R^2^ and Cox & Snell R^2^ statistics [26]. Using binary probit regression, we evaluated the association between the propensity score predicted by the logistic regression model and the risk of 50-day in-hospital mortality.

We also evaluated the association between plasma procalcitonin at baseline and 50-day in-hospital mortality using univariate and multivariable survival analyses. We estimated cumulative probabilities of survival using the Kaplan–Meier method. Survival time was calculated from the date of hospital admission. Surviving patients were censored at hospital discharge or the last known follow-up in the 50 days following hospital admission. Follow-up times were calculated and expressed in days. Univariate analysis was performed using the log-rank test to compare patient subgroups according to the ROC-defined threshold of plasma procalcitonin at baseline. The results were expressed as hazard ratios (HRs), 95% CIs, and the associated *P* values. In multivariable analyses, we assessed the association between the ROC-defined threshold of plasma procalcitonin at baseline and in-hospital mortality using Cox proportional hazards regression analysis with the forced entry or the stepwise method, with age, pathogen genera identified in blood cultures, C-reactive protein, and sepsis status at admission as covariates for adjustment. All variables with *P* < 0.2 were included in the model, and those with *P* < 0.05 were retained. The results are expressed as HRs, 95% CIs, and the associated *P* values. We assessed the Cox proportional hazards regression model discrimination between positive and negative cases using C-index statistics by calculating the area under the ROC curve of the prognostic indices generated [27].

In sensitivity analyses, we used quartile-transformed procalcitonin values to assess the association between this 4-class variable and 50-day in-hospital mortality. We assessed the association between crude rates of 50-day in-hospital mortality and procalcitonin quartiles using the chi-squared and chi-squared test for trend for univariate analysis and logistic regression in multivariable analysis, as reported above. We also assessed the association between procalcitonin quartiles and in-hospital mortality using the log-rank test and the log-rank test for trend for univariate analysis and Cox proportional hazards regression in multivariable analysis. All statistical analyses were conducted with JASP (version 0.14.1) and MedCalc (version 20.010) based on a two-sided type I error with an alpha level of 0.05.

## Results

### Description of the population

Between January 1, 2006, and December 31, 2012, 1593 patients were admitted to one of the healthcare facilities of the University Hospital of Nancy from home or through the emergency department and had positive blood cultures with concomitant procalcitonin assay. Among them, 838 had positive cultures from blood drawn within 48 hours of admission. After excluding patients with blood culture contaminations (*n* = 364), 474 patients were classified as having a CA-BSI. Among them, 452 had available follow-up data in the 50 days following hospital admission and were included in the analysis (Supplemental Fig. S1). The median age was 61 years (IQR, 31–74), and the proportion of males was 60.6% (274/452) (Table 1). Seventy percent (316/452) of patients had a bloodstream infection with *Staphylococcus aureus*, *Escherichia coli*, or *Streptococcus* spp. (Table 1). During the first 50 days following hospital admission, 88 patients died (19.5%; 95% CI, 15.8–23.1). The median duration of hospital stay in the whole cohort was 12 days (IQR, 6–22) and was significantly lower among patients in the 50-day mortality subgroup (5 days, [IQR, 1–15]) than among those without 50-day mortality (13 days [IQR, 7–23]).

**Table 1:** Characteristics of the 452 consecutive patients with community-acquired bloodstream infection included in the study

Characteristic	Whole study (***n*** = 452)		Patients with 50-day mortality (***n*** = 88)		Patients without 50-day mortality (***n*** = 364)		***P*** value
Age — N, median (IQR)	452	61 (31–74)	88	65 (55–78)	364	60 (22–72)	0.0002*
Male gender — n/N, % (95% CI)	274/452	60.6% (56.1–65.1)	54/88	61.4% (51.0–71.7)	220/364	60.4% (55.4–65.5)	0.90†
Procalcitonin (ng/mL) — N, median (IQR)	452	2.54 (0.60–11.75)	88	7.40 (1.94–19.88)	364	2.15 (0.490–10.57)	< 0.0001*
Sepsis at admission — n/N, % (95% CI)	92/452	20.4% (16.6–24.1)	16/88	18.2% (10.0–26.4)	76/364	20.9% (16.7–25.1)	0.57†
C-reactive protein (mg/L) — N, median (IQR)	326	138.9 (65.6–224.3)	59	166.5 (102.7–264.0)	267	130.0 (63.0–210.9)	0.006*
Lactates (mmol/L) — N, median (IQR)	203	1.7 (1.1–3.1)	54	2.5 (1.5–4.1)	149	1.6 (1.075–2.9)	0.002*
Cardiac troponin I (ng/mL) — N, median (IQR)	133	0.11 (0.04–0.43)	44	0.29 (0.07–1.80)	89	0.08 (0.04–0.23)	0.0003*
Gram staining, fungi — n/N, % (95% CI)							
Gram-positive	229/452	50.7% (46.0–55.3)	43/88	48.9% (38.2–59.5)	186/364	51.1% (45.9–56.3)	0.72†
Gram-negative	203/452	44.9% (40.3–49.5)	41/88	46.6% (36.0–57.2)	162/364	44.5% (39.4–49.6)	0.72†
Fungi	20/452	4.4% (2.5–6.3)	4/88	4.5% (0.1–8.9)	16/364	4.4% (2.28–6.5)	0.99†
Microorganisms — n/N, % (95% CI)							
*Staphylococcus aureus*	123/452	27.2% (23.1–31.3)	29/88	33.0% (22.9–43.0)	94/364	25.8% (21.3–30.3)	0.18†
*Escherichia coli*	117/452	25.9% (21.8–29.9)	20/88	22.7% (13.8–31.7)	97/364	26.6% (22.1–31.2)	0.45†
*Streptococcus* spp.	76/452	16.8% (13.4–20.3)	6/88	6.8% (1.5–12.2)	70/364	19.2% (15.2–23.3)	0.005†
*Enterococcus* spp.	30/452	6.6% (4.3–8.9)	8/88	9.1% (3–15.2)	22/364	6.0% (3.6–8.5)	0.30†
*Klebsiella* spp.	29/452	6.4% (4.2–8.7)	10/88	11.4% (4.6–18.1)	19/364	5.2% (2.9–7.5)	0.04†
*Pseudomonas* spp.	27/452	6.0% (3.8–8.2)	5/88	5.7% (0.7–10.6)	22/364	6.0% (3.6–8.5)	0.90†
*Fungi*	20/452	4.4% (2.5–6.3)	4/88	4.6% (0.1–9.0)	16/364	4.4% (2.3–6.5)	0.95†
*Enterobacter* spp.	15/452	3.3% (1.7–5)	5/88	5.7% (0.7–10.6)	10/364	2.8% (1.1–4.4)	0.17†
*Bacteroides* spp.	7/452	1.6% (0.4–2.7)	1/88	1.1% (0–3.4)	6/364	1.7% (0.3–3.0)	—‡
*Acinetobacter* spp.	5/452	1.1% (0.1–2.1)	0/88	0% (—)	5/364	1.4% (0.2–2.6)	—‡
*Citrobacter* spp.	3/452	0.7% (0–1.4)	0/88	0% (—)	3/364	0.8% (0–1.8)	—‡

*Note*. *IQR* interquartile range, 95% *CI*: 95% confidence interval, *spp*. species

*Wilcoxon Mann-Whitney test (patients with vs. without 50-day mortality)

†Chi-squared test or Fischer’s exact test, as appropriate (patients with vs. without 50-day mortality)

‡Low sample size

### Predictors of 50-day in-hospital mortality in univariate analysis

In univariate analysis, age, procalcitonin, C-reactive protein, lactate, and cardiac troponin I were significantly associated with an increased risk of 50-day in-hospital mortality. In contrast, blood culture positivity for *Streptococcus* spp. was significantly associated with a reduced risk of 50-day in-hospital mortality (Table 1). In the overall cohort, the proportion of patients with sepsis on admission was 20.4% (92/452). This proportion did not differ between patients with or without 50-day mortality (18.2% vs. 20.9%, *P* = 0.57) (Table 1). In univariate survival analysis, a troponin level at admission > 0.05 ng/mL (AUROC, 0.691; 95% CI, 0.592–0.778; *P* = 0.0001) and a lactate level at admission > 2.4 mmol/L (AUROC, 0.643; 95% CI, 0.559 to 0.722; *P* = 0.001) were significantly associated with an increased risk of in-hospital mortality (HR, 2.56; 95% CI, 1.33–4.89; *P* = 0.005 and HR, 3.14; 95% CI, 1.76–5.62; *P* = 0.0001, respectively) (Supplemental Fig. S2). However, cardiac troponin I and lactate were available in only 29% (203/542) and 45% (203/452) of the patients, respectively, and could not be used in multivariable analyses.

### Association between baseline procalcitonin level and 50-day in-hospital mortality

**Fig. 1:**
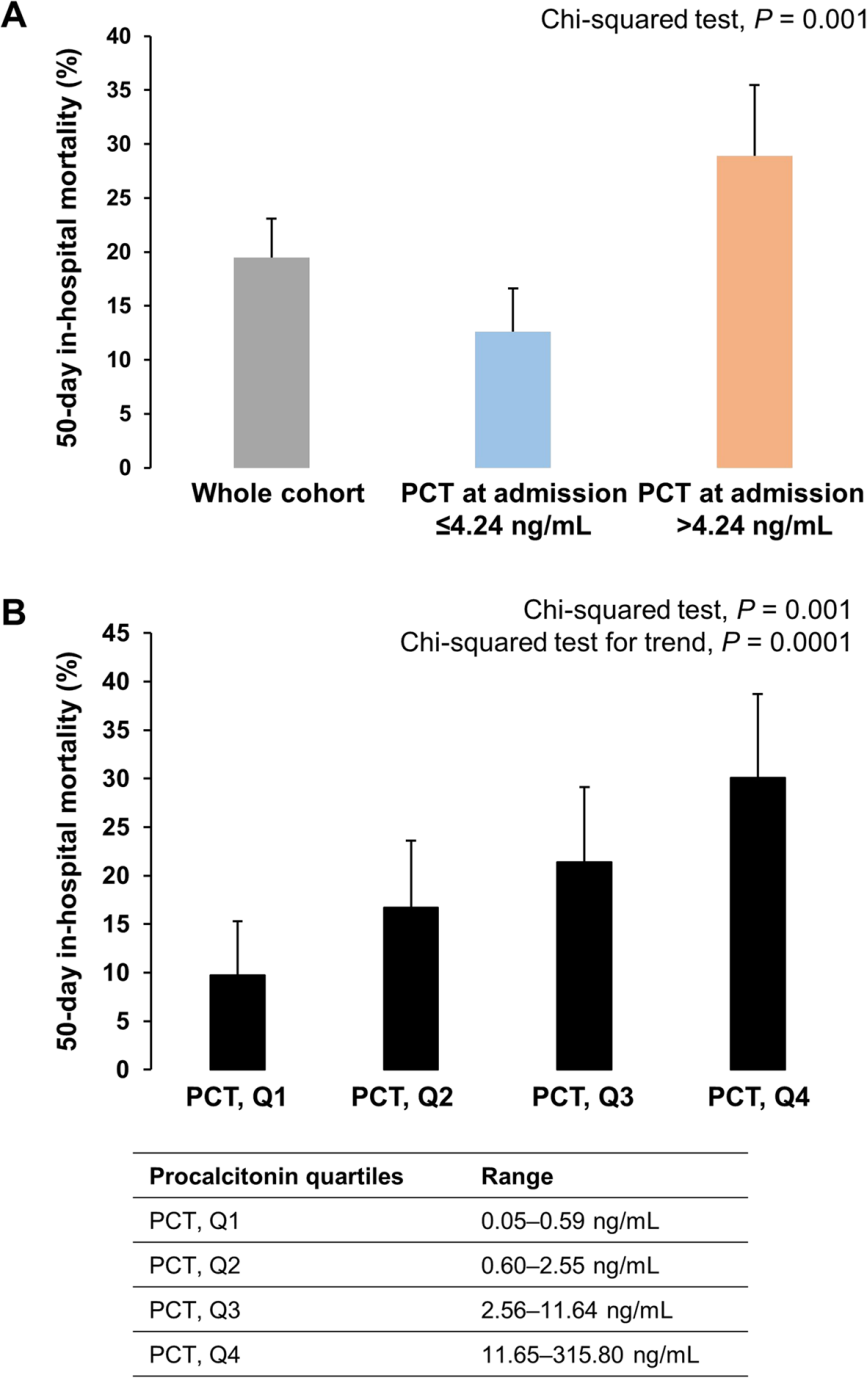
**A** 50-day in-hospital mortality rate in the whole cohort and according to baseline plasma procalcitonin threshold defined in receiver operating characteristic (ROC) analysis (> 4.24 ng/mL); **B** 50-day in-hospital mortality rate according to baseline plasma procalcitonin quartiles

The median procalcitonin value at baseline was significantly higher among patients in the 50-day mortality subgroup than among those without 50-day in-hospital mortality (7.40 ng/mL, IQR: 1.94–19.88 vs. 2.15 ng/mL, IQR: 0.49–10.57; *P* < 0.0001) (Table 1). In ROC analysis, procalcitonin at baseline was significantly associated with 50-day in-hospital mortality with an optimal threshold > 4.24 ng/mL (AUROC, 0.640; 95% CI, 0.578 to 0.700; *P* < 0.0001). The 50-day in-hospital mortality rate among patients with baseline procalcitonin > 4.24 ng/mL was 28.9% (95% CI, 22.4–35.5%) vs. 12.6% (95% CI, 8.6–16.6%) among those with a baseline procalcitonin ≤ 4.24 ng/mL (absolute difference, + 16.3%; 95% CI, 8.8–24.0%; *P* < 0.0001) (Fig. 1A). In multivariable logistic regression analysis, a baseline procalcitonin > 4.24 ng/mL was independently associated with an increased risk of 50-day in-hospital mortality (OR, 2.58; 95% CI, 1.57–4.25; *P* = 0.0002) (Table 2, Supplemental Table S1, and Supplemental Fig. S3). The independent association between a baseline procalcitonin level > 4.24 ng/mL and the risk of 50-day in-hospital mortality, in logistic regression analysis, was maintained even after adjusting for C-reactive protein (mg/L) and sepsis status at admission (OR, 2.63; 95% CI, 1.37–5.03; *P* = 0.004) (Supplemental Table S2).

**Table 2:** Association between plasma procalcitonin level at admission and all-cause 50-day mortality in multivariable logistic regression analysis

Covariate	Odds ratio	95% CI	***P*** value*	Percent of cases correctly classified	AUROC^a^ (95% CI)
**Model #1: Plasma PCT, ROC-defined threshold**	–			81%	0.721 (0.677–0.762)
Procalcitonin > 4.24 ng/mL	2.58	1.57 to 4.25	0.0002	–	–
Age (years)	1.02	1.01 to 1.03	0.0007	–	–
Pathogen genus, *Streptococcus*	0.52	0.20 to 1.30	0.16	–	–
Pathogen genus, *Staphylococcus*	1.59	0.91 to 2.78	0.10	–	–
Pathogen genus, *Klebsiella*	1.92	0.81 to 4.54	0.14	–	–
**Model #2: Plasma PCT quartiles (continuous)**	–			80%	0.711 (0.669–0.754)
Procalcitonin, quartiles (continuous)	1.47	1.17 to 1.85	0.001	–	–
Age (years)	1.02	1.01 to 1.03	0.0007	–	–
Pathogen genus, *Streptococcus*	0.52	0.21 to 1.31	0.17	–	–
Pathogen genus, *Staphylococcus*	1.55	0.89 to 2.70	0.12	–	–
Pathogen genus, *Klebsiella*	1.93	0.82 to 4.55	0.13	–	–
**Model #3: Plasma PCT, 4th vs. 1st to 3rd quartiles**	–			81%	0.702 (0.658–0.744)
Procalcitonin, 4th quartile	2.12	1.26 to 3.54	0.004	–	–
Age (years)	1.02	1.01 to 1.03	0.0004	–	–
Pathogen genus, *Streptococcus*	0.49	0.20 to 1.24	0.13	–	–
Pathogen genus, *Staphylococcus*	1.54	0.89 to 2.69	0.13	–	–
Pathogen genus, *Klebsiella*	2.07	0.88 to 4.88	0.10	–	–
**Model #4: Plasma PCT, 4th vs. 1st quartile**	–			80%	0.723 (0.660–0.780)
Procalcitonin, 4th quartile	3.30	1.53 to 7.12	0.002	–	–
Age (years)	1.01	1.00 to 1.02	0.08	–	–
Pathogen genus, *Streptococcus*	0.47	0.13 to 1.72	0.25	–	–
Pathogen genus, *Staphylococcus*	1.35	0.62 to 2.97	0.45	–	–
Pathogen genus, *Klebsiella*	1.93	0.48 to 7.78	0.36	–	–

*Note*. *AUROC* area under the receiver operating characteristic curve, *PCT* procalcitonin

*Logistic regression using the forced entry method

^a^AUROC of the prognostic indices generated by the logistic regression model to discriminate between positive and negative cases

In the survival analysis, the mean survival time in the patients without in-hospital mortality was 37 days (95% CI, 35–39 days). The survival probabilities in patients without in-hospital mortality at Days 10, 20, 30, and 40 were 85.4% (standard error [SE], 1.8), 78.7% (SE, 2.4), 69.7% (SE, 3.5), and 63.4% (SE, 4.4), respectively (Supplemental Fig. S4A). Patients with a baseline procalcitonin > 4.24 ng/mL (ROC-defined threshold) had a significantly increased risk of in-hospital mortality (HR, 2.28; 95% CI, 1.49–3.49; *P* = 0.0001) (Supplemental Fig. S4B and Supplemental Tables S3 and S4). In multivariable Cox proportional hazard regression analysis, a baseline procalcitonin > 4.24 ng/mL was independently associated with an increased risk of in-hospital mortality (HR, 2.01; 95% CI, 1.30–3.11; *P* = 0.002) (Table 3). The independent association between a baseline procalcitonin > 4.24 ng/mL and the risk of in-hospital mortality in Cox regression analysis was maintained even after adjusting for C-reactive protein (mg/L) and sepsis status at admission (HR, 2.11; 95% CI, 1.20–3.70; *P* = 0.01) (Supplemental Table S5).

**Table 3:** Association between plasma procalcitonin level at admission and all-cause in-hospital mortality in multivariable Cox proportional-hazards regression

Covariate	Hazard ratio	95% CI	***P*** value*	AUROC^a^ (95% CI)
**Model #1: Plasma PCT, ROC-defined threshold**	–			0.688 (0.643–0.730)
Procalcitonin > 4.24 ng/mL	2.01	1.30–3.11	0.002	–
Age (years)	1.01	1.00–1.02	0.02	–
Pathogen genus, *Streptococcus*	0.50	0.21–1.18	0.12	–
Pathogen genus, *Staphylococcus*	1.13	0.70–1.83	0.62	–
Pathogen genus, *Klebsiella*	1.57	0.79–3.13	0.20	–
**Model #2: Plasma PCT quartiles**	–			0.708 (0.664–0.750)
Procalcitonin, quartiles	1.31	1.07–1.60	0.008	–
Age (years)	1.01	1.00–1.02	0.02	–
Pathogen genus, *Streptococcus*	0.51	0.22–1.22	0.13	–
Pathogen genus, *Staphylococcus*	1.11	0.69–1.80	0.66	–
Pathogen genus, *Klebsiella*	1.61	0.81–3.21	0.18	–
**Model #3: Plasma PCT, 4th vs. 1st to 3rd quartiles**	–			0.699 (0.655–0.741)
Procalcitonin, 4th quartile	1.62	1.05–2.50	0.03	–
Age (years)	1.01	1.00–1.02	0.01	–
Pathogen genus, *Streptococcus*	0.49	0.21–1.16	0.10	–
Pathogen genus, *Staphylococcus*	1.13	0.70–1.83	0.61	–
Pathogen genus, *Klebsiella*	1.71	0.86–3.41	0.13	–
**Model #4: Plasma PCT, 4th vs. 1st quartile**	–			0.716 (0.652–0.774)
Procalcitonin, 4th quartile	2.44	1.21–4.94	0.01	–
Age (years)	1.01	0.99–1.02	0.31	–
Pathogen genus, *Streptococcus*	0.50	0.15–1.70	0.27	–
Pathogen genus, *Staphylococcus*	0.98	0.50–1.93	0.95	–
Pathogen genus, *Klebsiella*	1.51	0.52–4.39	0.45	–

*Note*. *AUROC* area under the receiver operating characteristic curve, *PCT* procalcitonin

*Cox proportional-hazards regression using the forced entry method

^a^AUROC of the prognostic indices generated by the Cox proportional-hazards regression model to discriminate between positive and negative cases

### Sensitivity analyses

We performed a post hoc sensitivity analysis using baseline procalcitonin quartiles, considered a 4-class variable (Supplemental Table S6). In patients in the 1st (range, 0.05–0.59 ng/mL), 2nd (range, 0.60–2.55 ng/mL), 3rd (range, 2.56–11.64 ng/mL), and 4th (range, 11.65–315.80 ng/mL) procalcitonin quartiles at baseline, the 50-day in-hospital mortality rates were 9.7% (4.18–15.3%), 16.7% (9.7–23.6%), 21.4% (13.7–29.1%), and 30.1% (21.5–38.7%), respectively (Chi-squared test, *P* = 0.001; Chi-squared test for trend, *P* = 0.0001). When compared to the 1st procalcitonin quartile, patients in the 2nd, 3rd, and 4th quartiles had the following absolute differences in 50-day in-hospital mortality: + 7% (95% CI, − 1.9–16.0%), + 11.7% (2.2–21.216%), and + 20.4% (95% CI, 10.1–30.3%), respectively. In multivariable logistic regression analysis, baseline procalcitonin quartiles were independently associated with 50-day in-hospital mortality with an OR of 1.47 (95% CI, 1.17–1.85; *P* = 0.001) per quartile increment (OR, 2.12; 95% CI, 1.26–3.54; *P* = 0.004 for the comparison between 4th and 1st to 3rd quartiles and OR, 3.30; 95% CI, 1.53–7.12; *P* = 0.002 for the comparison between the 4th and the 1st quartiles). These results were similar after adjusting for C-reactive protein (mg/L) and sepsis status at admission (OR, 1.38; 95% CI, 1.01 to 1.87; *P* = 0.04; OR, 1.92; 95% CI, 1.02–3.60; *P* = 0.04; and OR, 3.41; 95% CI, 1.33 to 8.73; *P* = 0.01, respectively) (Supplemental Table S2).

**Fig. 2:**
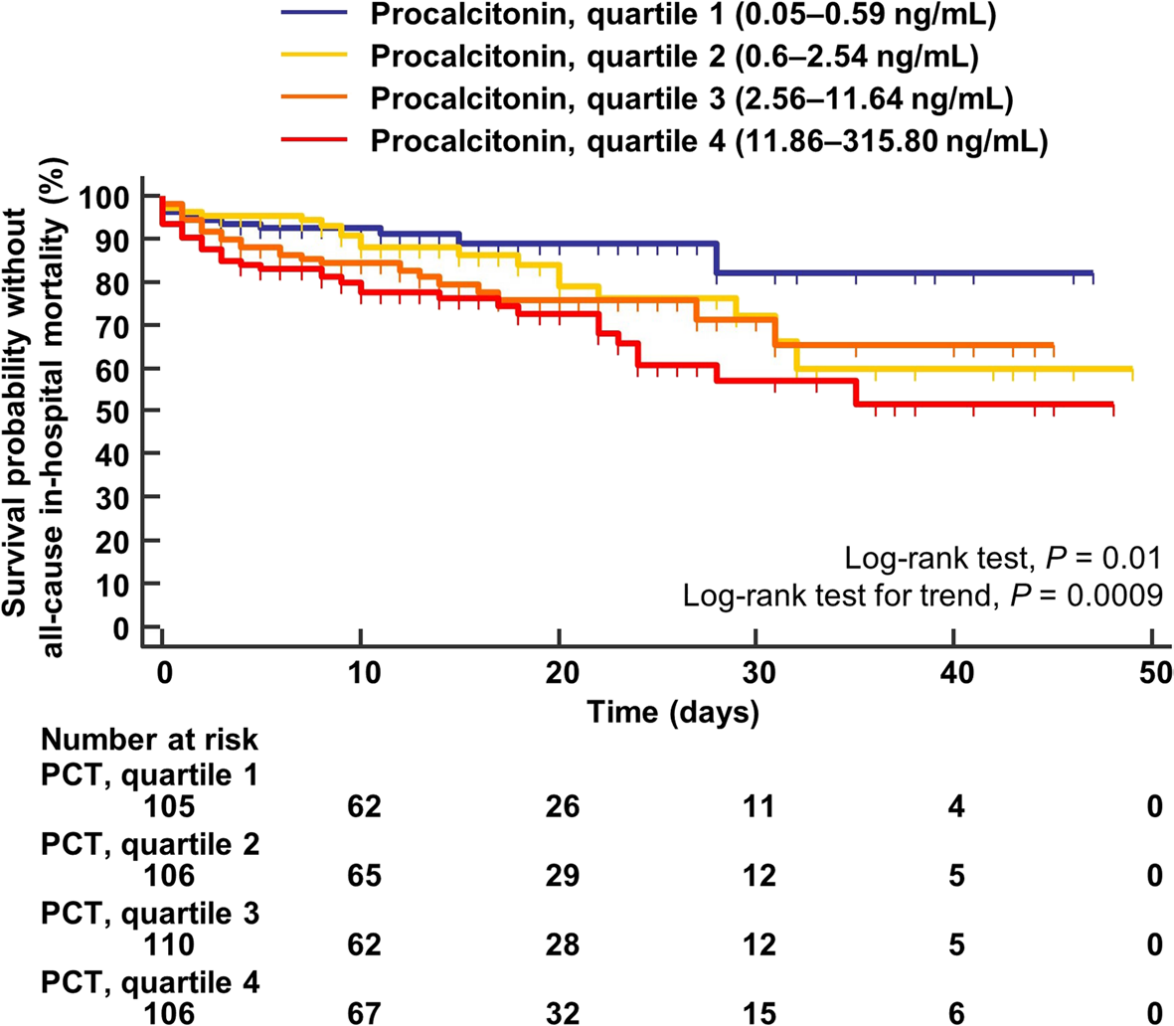
Kaplan–Meier analysis reporting the probability of survival without in-hospital mortality in the 452 studied patients according to baseline plasma procalcitonin quartiles

In survival analysis, baseline procalcitonin quartiles were significantly associated with in-hospital mortality (log-rank test, *P* = 0.01; log-rank test for trend, *P* = 0.0009) (Supplemental Table S7 and Fig. 2). The univariate HRs for in-hospital mortality of patients in the 2nd, 3rd, and 4th procalcitonin quartiles, when compared to those in the 1st quartile, were 1.62 (95% CI, 0.89–2.93), 2.10 (95% CI, 1.15–3.81), and 2.87 (95% CI, 1.58–5.19), respectively (Supplemental Table S8). In multivariable Cox proportional hazard regression analysis, baseline procalcitonin quartiles were independently associated with an increased risk of in-hospital mortality (HR, 1.31; 95% CI, 1.07–1.60; *P* = 0.008) (Table 2). Patients in the 4th quartile of procalcitonin had an increased risk of in-hospital mortality when compared to those in the 1st to 3rd quartiles (HR, 1.62; 95% CI, 1.05–2.50; *P* = 0.03) or those in the 1st quartile only (HR, 2.44; 95% CI, 1.21–4.94; *P* = 0.01) (Table 2). These results were similar after adjusting for C-reactive protein (mg/L) and sepsis status at admission (HR, 1.35; 95% CI, 1.06–1.72; *P* = 0.02; HR, 1.73; 95% CI, 1.13–2.66; *P* = 0.01; and HR, 3.08; 95% CI, 1.32–7.22; *P* = 0.01, respectively) (Supplemental Table S5).

## Discussion

We report the most comprehensive study to evaluate the usefulness of procalcitonin at admission for predicting 50-day in-hospital mortality among patients with CA-BSIs and provide the first evidence regarding its usefulness as a risk-stratifying biomarker. The crude 50-day mortality rate was 28.9% in patients with an admission procalcitonin level > 4.24 ng/mL and was 2.3-fold lower (12.6%) among patients with a procalcitonin level ≤ 4.24 ng/mL. Sensitivity analyses and dose-response probit regression confirmed a progressive increase in the risk of 50-day in-hospital mortality across procalcitonin quartiles, with a 47% increase in mortality risk for each procalcitonin quartile increment at admission. These results were corroborated by multivariable survival analyses, which reported a hazard ratio of 1.31 for in-hospital mortality risk per each procalcitonin quartile increment at admission.

To date, no study has investigated the usefulness of plasma procalcitonin at admission to predict in-hospital mortality among patients with CA-BSIs. Only one study assessed the association between procalcitonin and mortality among 239 patients with community-acquired bacteremia [28]. However, in this study, no multivariable analysis was carried out to estimate the risk after adjusting for potential confounding factors, and no survival analyses were performed.

Bloodstream infections represent a leading cause of infectious disease morbidity and mortality worldwide [9–15]. Timely treatment affects both the financial burden and the clinical outcome of bloodstream infections, significantly benefiting survival [29, 30]. In this setting, the availability of a fast peripheral blood prognostic biomarker could help stratify the risk of mortality associated with bloodstream infections and adapt optimized diagnostic and therapeutic strategies. Procalcitonin is a sepsis-related biomarker that is routinely assayed in an automated manner with defined quality standards and a run time of less than 30 minutes [19, 31–33]. In healthy subjects, procalcitonin blood concentrations are extremely low. However, they can increase 1000-fold following bacterial or parasitic infections [19, 34–36]. Following endotoxin treatment of baboons, it has been shown that procalcitonin is produced by several tissues, mainly the liver and kidneys, as soon as 6 h after the injection [34, 37]. Several studies have evaluated the usefulness of procalcitonin as a prognostic marker in patients with sepsis. A meta-analysis of 23 studies concluded that an elevated procalcitonin concentration was strongly associated with all-cause mortality in patients with sepsis, with a pooled relative risk of 2.60 [38]. However, the cutoff values used were highly heterogeneous, which precludes any possibility of translating the results of this meta-analysis into clinical practice [38].

Community-acquired infections contribute to 70% of cases of sepsis, which is associated with a mortality rate of up to 50% [1–3]. In 2001, early goal-directed therapy was shown to allow for early diagnosis and treatment of sepsis in 2002 through the Surviving Sepsis Campaign and the adoption of the Early Management Bundle for Severe Sepsis/Septic Shock (SEP-1) in 2015 [17]. Nevertheless, three randomized trials failed to demonstrate the benefit of early goal-directed therapy in patients with sepsis. In a retrospective cohort study that assessed the association of the early management bundle for severe sepsis/septic shock (SEP-1) with mortality and organ dysfunction, among 4108 patients with community-onset sepsis, serum lactate level testing within 3 hours of time 0 was associated with reduced mortality [1]. Similarly, our results prove that an initial procalcitonin assay could represent an additional tool as a risk-stratifying biomarker among patients with CA-BSIs. This assay can be conducted in a short time and is compatible with rapid decision-making, particularly in the setting of sepsis management, according to current guidelines to prevent unnecessary deaths and disability [6, 17].

Our study has several strengths. First, we report the first evidence regarding the usefulness of procalcitonin as a risk-stratifying biomarker in patients with CA-BSIs. Second, we used standardized data from the Nancy Biochemical Database [19–22]. This prospectively maintained electronic database increases the biological findings of consecutive patients hospitalized in all healthcare departments of medicine, surgery, and obstetrics at the University Hospital of Nancy. Third, in our study, procalcitonin was measured using the BRAHMS PCT-sensitive Kryptor method, which was used in most studies investigating procalcitonin use and offered a lower detection limit of 0.02 ng/mL and functional assay sensitivity of 0.06 ng/mL [39]. We acknowledge several potential limitations of the study that should be considered in interpreting our results. First, our results are based on a retrospective, observational cohort study and should be further validated in independent prospective cohorts. However, we report the most comprehensive study to address the knowledge gap regarding the usefulness of procalcitonin as a risk-stratifying biomarker in patients with CA-BSIs. Second, we excluded patients with peripheral blood culture contamination based on a widely accepted definition without formally excluding the risk of true bacteremia for classic contaminant microorganisms. However, true bacteremias for classical contaminants are mainly observed among patients with healthcare-associated and hospital-acquired bloodstream infections rather than those with CA-BSIs [7]. Furthermore, we recently showed that patients with blood culture contamination had a plasma procalcitonin level of < 0.1 ng/mL [40]. Third, given the study design and the architecture of the Nancy Biochemical Database, we could not determine the final diagnosis or the source of the infection in the patients included in the study. Fourth, our study did not assess the Sepsis-related Organ Failure (SOFA) and Acute Physiologic Assessment and Chronic Health Evaluation Scoring System II (APACHE II) scores to refine the prognostic performance of procalcitonin for mortality prediction since these data were not available in the Nancy Biochemical Database. In a prospective, observational study on 53 patients with septic shock, procalcitonin levels were correlated with the APACHE II score, and the decrease in inaugural procalcitonin was associated with a higher probability of survival [41].

In conclusion, we report the most comprehensive study that provides proof-of-concept evidence of the usefulness of plasma procalcitonin as a risk-stratifying biomarker for predicting 50-day in-hospital mortality among patients with CA-BSIs. Future prospective studies should investigate the added value of procalcitonin in assessing patients with CA-BSIs to improve risk prediction and provide personalized therapeutic management.

## Supplementary Information

**Additional file 1: Supplemental methods**. **Supplemental Table S1.** Probit regression analysis for the propensity score predicted by the logistic regression model as the dose variable and the risk of 50-day in-hospital mortality as the response variable. **Supplemental Table 2.** Association between plasma procalcitonin level at admission and all-cause 50-day mortality in multivariable logistic regression analysis. **Supplemental Table S3.** Kaplan-Meier analysis reporting the probability of survival without in-hospital mortality according to the ROC-defined plasma procalcitonin threshold (> 4.24 ng/mL) at baseline. **Supplemental Table S4.** Hazard ratios and 95% confidence interval for the pairwise comparison of survival probabilities without in-hospital mortality according to the ROC-defined plasma procalcitonin threshold (> 4.24 ng/mL) at baseline. **Supplemental Table S5.** Association between plasma procalcitonin level at admission and all-cause in-hospital mortality in multivariable Cox proportional-hazards regression. **Supplemental Table S6.** Distribution of procalcitonin values according to procalcitonin quartiles. **Supplemental Table S7.** Kaplan-Meier analysis reporting the probability of survival without in-hospital mortality in the 452 studied patients according to baseline plasma procalcitonin quartiles. **Supplemental Table S8.** Hazard ratios and 95% confidence interval for the pairwise comparison of survival probabilities without in-hospital mortality according to baseline procalcitonin quartiles. **Supplemental Figure S1.** Flow diagram of patient selection. **Supplemental Figure S2.** (A) Kaplan-Meier analysis reporting the probability of survival without in-hospital mortality according to the ROC-defined plasma troponin threshold (> 0.05 ng/mL) at baseline; (B) Kaplan-Meier analysis reporting the probability of survival without in-hospital mortality according to the ROC-defined plasma lactates threshold (> 4.24 ng/mL) at baseline. **Supplemental Figure S3.** Probit sigmoid dose-response curve showing the propensity score predicted by the logistic regression model as the dose variable and the risk of 50-day in-hospital mortality as the response variable. The red line shows the probability and corresponding dose. The dashed curves represent the 95% confidence interval for the respective dose. The dose and 95% confidence interval corresponding with a particular probability are taken from a horizontal line at that probability level. **Supplemental Figure S4.** (A) Kaplan-Meier ainalysis reporting the probability of survival without in-hospital mortality in the 452 studied patients. The dashed lines represent the 95% confidence interval of the survival probabilities; (B) Kaplan-Meier analysis reporting the probability of survival without in-hospital mortality according to the ROC-defined plasma procalcitonin threshold (> 4.24 ng/mL) at baseline.

## Data Availability

Anonymized patient data are available for use in collaborative studies to researchers upon reasonable request (abderrahim.oussalah@univ-lorraine.fr). Data will be provided following the review and approval of a research proposal (including a statistical analysis plan) and the completion of a data-sharing agreement. The University Hospital of Nancy IRB will assess responses to the request for the raw data.

## Abbreviations

95% CI: 95% confidence interval
AUROC: Area under the receiver operating characteristic curve
CA-BSIs: Community-acquired bloodstream infections
HR: Hazard ratio
IQR: Interquartile range (25–75th percentiles)
OR: Odds ratios
ROC analysis: Receiver operating characteristic analysis
