# Supplementary material for: Usefulness of procalcitonin at admission as a risk-stratifying biomarker for 50-day in-hospital mortality among patients with community-acquired bloodstream infection: an observational cohort study
Source: Biomark Res. 2023 Jan 17;11:4. doi: 10.1186/s40364-023-00450-3 (PMC9843889; doi:10.1186/s40364-023-00450-3)

**Usefulness of Procalcitonin at Admission as a Risk-Stratifying Biomarker for 50-Day In-Hospital Mortality Among Patients With Community-Acquired Bloodstream Infection: An Observational Cohort Study**

Abderrahim Oussalah, MD, PhD^1,2^; Jonas Callet, MD^1^; Nejla Aissa, MD^3^; Anne-Elisabeth Manteaux, PharmD^3^; Nathalie Thilly, PharmD, PhD^4^; Nicolas Jay, MD, PhD^5,6^; Jean-Louis Guéant MD, DSc, AGAF^1,2^; and Alain Lozniewski, MD, PhD^2,7^

1. Department of Molecular Medicine, Division of Biochemistry, Molecular Biology, and Nutrition, University Hospital of Nancy, Vandoeuvre-lès-Nancy, F-54000, France.

2. University of Lorraine, INSERM UMR_S 1256, Nutrition, Genetics, and Environmental Risk Exposure (NGERE), Faculty of Medicine of Nancy, Vandoeuvre-lès-Nancy, F-54000, France.

3. Department of Microbiology, University Hospital of Nancy, Vandoeuvre-lès-Nancy, F-54000, France.

4. University of Lorraine, University Hospital of Nancy, Department of Methodology, Promotion and Investigation, F-54000 Nancy, France.

5. Department of Medical Informatics, University Hospital, Vandoeuvre-lès-Nancy, France.

6. Orpailleur, LORIA UMR 7503, Vandoeuvre-lès-Nancy, F-54000, France.

7. EA7300, Stress Immunity Pathogens Laboratory, Faculty of Medicine of Nancy, University of Lorraine, Vandoeuvre-lès-Nancy, F-54000, France.

**Corresponding Author:**

Prof. Abderrahim Oussalah, MD, PhD

Department of Molecular Medicine, Division of Biochemistry, Molecular Biology, and Nutrition, University Hospital of Nancy, and INSERM UMR_S 1256, Nutrition, Genetics, and Environmental Risk Exposure (NGERE), Faculty of Medicine of Nancy, 9 Avenue de la Forêt de Haye, F-54000, Nancy, France.

Telephone: +33 3 83 15 36 29

Fax: +33 3 83 15 35 91

E-mail address: [abderrahim.oussalah@univ-lorraine.fr](mailto:abderrahim.oussalah@univ-lorraine.fr)

**SUPPLEMENTAL METHODS**

**Data collected and sources**

The following data were collected for the study: patient identification number; patient’s age at hospital admission; sex; date and time of blood sampling; patient’s healthcare department; Gram-stain results; identification results of blood culture isolates; biochemical markers at hospital admission including, procalcitonin (ng/mL), cardiac troponin I (ng/mL), C-reactive protein (mg/L), and lactates (mmol/L); the occurrence of death; date of death; date of hospital admission and discharge; date of last known follow-up after hospital admission. For each patient, procalcitonin and microbiological data were systematically collected in the Nancy Biochemical Database and extracted for the study’s purposes using the GLIMS general laboratory information management system, version 8.11.6 (MIPS France S.a.r.l., Paris, France). Data regarding in-hospital mortality were obtained from the Department of Medical Informatics of the University Hospital of Nancy.

**Procalcitonin assay and blood culture**

As previously reported, plasma procalcitonin concentration was measured using an automated immunofluorescent assay in human plasma samples (Brahms PCT sensitive KRYPTOR kit for Brahms KRYPTOR, Hennigsdorf, Germany) according to the supplier’s protocol. Blood culture bottles (Bactec^®^ Plus Aerobic and Bactec^®^ F Lytic Anaerobic, Becton Dickinson, Le Pont de Claix, France) were incubated for at least five days using the BD Bactec^™^ 9240 blood culture system. If no bacterial growth was detected within the incubation period, blood culture was considered negative. Aliquots taken from each positive bottle were Gram-stained and subcultured on appropriate culture media for subsequent analysis. Identification of microorganisms was performed using phenotypic methods, i.e., conventional methods, the Vitek 2 system (bioMérieux, Marcy L’Étoile, France), and from July 2012, the Vitek MS system (bioMérieux), and16S rRNA gene sequencing, if necessary.

**SUPPLEMENTAL TABLES**

**Supplemental Table S1. Probit regression analysis for the propensity score predicted by the logistic regression model as the dose variable and the risk of 50-day in-hospital mortality as the response variable.**

| **Probability** | **Propensity score** | **95% Confidence interval** | |
| --- | --- | --- | --- |
| 0.010 | -0.18648 | -0.39612 | -0.077764 |
| 0.020 | -0.11143 | -0.28544 | -0.020034 |
| 0.025 | -0.085605 | -0.24749 | -0.000041033 |
| 0.05 | 0.0011503 | -0.12081 | 0.067963 |
| 0.10 | 0.10117 | 0.021855 | 0.14975 |
| 0.20 | 0.22229 | 0.17789 | 0.26552 |
| 0.25 | 0.26831 | 0.22698 | 0.31968 |
| 0.50 | 0.45401 | 0.38702 | 0.57635 |
| 0.75 | 0.63971 | 0.53125 | 0.84883 |
| 0.80 | 0.68572 | 0.56645 | 0.91689 |
| 0.90 | 0.80684 | 0.65867 | 1.09646 |
| 0.95 | 0.90686 | 0.73453 | 1.24506 |
| 0.975 | 0.99362 | 0.80019 | 1.37407 |
| 0.980 | 1.01944 | 0.81972 | 1.41249 |
| 0.990 | 1.09449 | 0.87643 | 1.52419 |

**Supplemental Table 2. Association between plasma procalcitonin level at admission and all-cause 50-day mortality in multivariable logistic regression analysis**

| **Covariate** | **Odds ratio** | **95% CI** | ***P*-value** | **Percent of**  **cases correctly**  **classified** | **AUROC**‡  **(95% CI)** |
| --- | --- | --- | --- | --- | --- |
| **Model #1: Plasma PCT, ROC-defined threshold***  **(n = 326)** | — | | | 83% | 0.763 (0.713 to 0.808) |
| Procalcitonin > 4.24 ng/mL | 2.63 | 1.37 to 5.03 | 0.004 | — | — |
| Age (years) | 1.03 | 1.01 to 1.05 | 0.0006 | — | — |
| Pathogen genus, *Streptococcus* | 0.36 | 0.10 to 1.29 | 0.12 | — | — |
| Pathogen genus, *Staphylococcus* | 1.99 | 0.98 to 4.03 | 0.06 | — | — |
| Pathogen genus, *Klebsiella* | 2.02 | 0.76 to 5.35 | 0.16 | — | — |
| C-reactive protein (mg/L) | 1.00 | 0.99 to 1.00 | 0.57 | — | — |
| Sepsis at admission | 1.04 | 0.48 to 2.24 | 0.92 | — | — |
|  |  |  |  |  |  |
| **Model #2: Plasma PCT quartiles (continuous)***  **(n = 326)** | — | | | 82% | 0.743 (0.692–0.790) |
| Procalcitonin, quartiles (continuous) | 1.38 | 1.01 to 1.87 | 0.04 | — | — |
| Age (years) | 1.03 | 1.01 to 1.05 | 0.0006 | — | — |
| Pathogen genus, *Streptococcus* | 0.36 | 0.10 to 1.31 | 0.12 | — | — |
| Pathogen genus, *Staphylococcus* | 1.94 | 0.97 to 3.91 | 0.06 | — | — |
| Pathogen genus, *Klebsiella* | 2.07 | 0.79 to 5.43 | 0.14 | — | — |
| C-reactive protein (mg/L) | 1.00 | 0.99 to 1.00 | 0.42 | — | — |
| Sepsis at admission | 1.01 | 0.47 to 2.17 | 0.98 | — | — |
|  |  |  |  |  |  |
| **Model #3: Plasma PCT, 4^th^ vs. 1^st^–3^rd^ quartiles**  **(n = 326)** | — | | | 82% | 0.714 (0.662–0.763) |
| Procalcitonin, 4^th^ quartile | 1.92 | 1.02–3.60 | 0.04 | — | — |
| Age (years) | 1.03 | 1.01–1.05 | 0.0003 | — | — |
| Pathogen genus, *Streptococcus* | 0.26 | 0.08–0.89 | 0.03 | — | — |
| Pathogen genus, *Staphylococcus* | Not retained in the logistic regression model | | | — | — |
| Pathogen genus, *Klebsiella* | Not retained in the logistic regression model | | | — | — |
| C-reactive protein (mg/L) | Not retained in the logistic regression model | | | — | — |
| Sepsis at admission | Not retained in the logistic regression model | | | — | — |
| **Model #4: Plasma PCT, 4^th^ vs. 1^st^ quartile**  **(n = 158)** | — | | | 82% | 0.721 (0.644–0.789) |
| Procalcitonin, 4^th^ quartile | 3.41 | 1.33 to 8.73 | 0.01 | — | — |
| Age (years) | 1.02 | 1.00 to 1.04 | 0.02 | — | — |
| Pathogen genus, *Streptococcus* | Not retained in the logistic regression model | | | — | — |
| Pathogen genus, *Staphylococcus* | Not retained in the logistic regression model | | | — | — |
| Pathogen genus, *Klebsiella* | Not retained in the logistic regression model | | | — | — |
| C-reactive protein (mg/L) | Not retained in the logistic regression model | | | — | — |
| Sepsis at admission | Not retained in the logistic regression model | | | — | — |

Note. AUROC: area under the receiver operating characteristic curve; PCT: procalcitonin.

* Logistic regression using the forced entry method.

† Logistic regression using the stepwise method.

‡ AUROC of the prognostic indices generated by the logistic regression model to discriminate between positive and negative cases.

**Supplemental Table S3. Kaplan-Meier analysis reporting the probability of survival without in-hospital mortality according to the ROC-defined plasma procalcitonin threshold (> 4.24 ng/mL) at baseline.**

|  | **Whole study**  **(n = 452)** | | **Procalcitonin ≤ 4.24 ng/mL**  **(n = 262)** | | **Procalcitonin > 4.24 ng/mL**  **(n = 190)** | |
| --- | --- | --- | --- | --- | --- | --- |
| **Survival time**  **(days)** | **Probability of**  **survival (%)** | **SE (%)** | **Probability of**  **survival (%)** | **SE (%)** | **Probability of**  **survival (%)** | **SE (%)** |
| 0 | 96.5 | 0.869 | 97.3 | 0.996 | 95.3 | 1.54 |
| 1 | 94.2 | 1.1 | 96.1 | 1.2 | 91.6 | 2.01 |
| 2 | 92.4 | 1.26 | 94.9 | 1.37 | 88.9 | 2.27 |
| 3 | 91 | 1.36 | 94.5 | 1.43 | 86.3 | 2.5 |
| 4 | 90.3 | 1.41 | — | — | 84.7 | 2.62 |
| 5 | 89.8 | 1.45 | 94.1 | 1.48 | 84.1 | 2.66 |
| 6 | 89.3 | 1.48 | — | — | 83 | 2.74 |
| 7 | 88.7 | 1.52 | 93.6 | 1.56 | 82.4 | 2.79 |
| 8 | 87.6 | 1.61 | 93.1 | 1.63 | 80.5 | 2.93 |
| 9 | 86.7 | 1.67 | 91.9 | 1.79 | 79.9 | 2.97 |
| 10 | 85.4 | 1.76 | 90.8 | 1.96 | 78.5 | 3.08 |
| 11 | 85.1 | 1.79 | 90.2 | 2.04 | — | — |
| 12 | 84.7 | 1.82 | — | — | 77.7 | 3.14 |
| 13 | 84.3 | 1.85 | 89.4 | 2.14 | — | — |
| 14 | 83.5 | 1.93 | — | — | 75.8 | 3.33 |
| 15 | 82.6 | 2.01 | 87.8 | 2.41 | — | — |
| 16 | 82.1 | 2.06 | — | — | 74.8 | 3.45 |
| 17 | 81 | 2.16 | — | — | 72.6 | 3.67 |
| 18 | 80 | 2.26 | 86.7 | 2.6 | 71.5 | 3.78 |
| 19 | — | — | — | — | — | — |
| 20 | 78.7 | 2.39 | 84.3 | 3.02 | — | — |
| 21 | — | — | — | — | — | — |
| 22 | 76.6 | 2.61 | 83 | 3.25 | 68.7 | 4.13 |
| 23 | 75.9 | 2.69 | — | — | 67.2 | 4.3 |
| 24 | 74.3 | 2.86 | — | — | 63.9 | 4.68 |
| 25 | — | — | — | — | — | — |
| 26 | — | — | — | — | — | — |
| 27 | 73.3 | 3 | — | — | 61.8 | 4.98 |
| 28 | 70.9 | 3.33 | 80.8 | 3.86 | 59.4 | 5.33 |
| 29 | 69.7 | 3.49 | 78.3 | 4.45 | — | — |
| 30 | — | — | — | — | — | — |
| 31 | 66.9 | 3.87 | 75.3 | 5.2 | 56.9 | 5.65 |
| 32 | 65.3 | 4.1 | 71.9 | 5.99 | — | — |
| 33 | — | — | — | — | — | — |
| 35 | 63.4 | 4.41 | — | — | 53.6 | 6.23 |
| 50 | — | — | — | — | — | — |

Note. SE: standard error.

**Comparison of survival curves (Log-rank test)**

| Chi-squared | 14.4145 |
| --- | --- |
| DF | 1 |
| Significance | P = 0.0001 |

**Supplemental Table S4. Hazard ratios and 95% confidence interval for the pairwise comparison of survival probabilities without in-hospital mortality according to the ROC-defined plasma procalcitonin threshold (> 4.24 ng/mL) at baseline.**

|  | **Procalcitonin**  **≤ 4.24 ng/mL** | **Procalcitonin**  **> 4.24 ng/mL** |
| --- | --- | --- |
| **Procalcitonin**  **≤ 4.24 ng/mL** | — | 2.28 1.49–3.49 |
| **Procalcitonin**  **> 4.24 ng/mL** | 0.44 0.29–0.67 | — |

**Supplemental Table S5. Association between plasma procalcitonin level at admission and all-cause in-hospital mortality in multivariable Cox proportional-hazards regression**

| **Covariate** | **Hazard ratio** | **95% CI** | ***P*-value** | **AUROC†**  **(95% CI)** |
| --- | --- | --- | --- | --- |
| **Model #1: Plasma PCT, ROC-defined threshold***  **(n = 326)** | — | | | 0.683 (0.624–0.742) |
| Procalcitonin > 4.24 ng/mL | 2.11 | 1.20–3.70 | 0.01 | — |
| Age (years) | 1.02 | 1.01–1.04 | 0.004 | — |
| Pathogen genus, *Streptococcus* | 0.35 | 0.11–1.18 | 0.09 | — |
| Pathogen genus, *Staphylococcus* | 1.41 | 0.77–2.58 | 0.27 | — |
| Pathogen genus, *Klebsiella* | 1.70 | 0.77–3.76 | 0.19 | — |
| C-reactive protein (mg/L) | 1.00 | 0.99–1.00 | 0.97 | — |
| Sepsis at admission | 1.13 | 0.60–2.12 | 0.71 | — |
| **Model #2: Plasma PCT quartiles‡**  **(n = 326)** | — | | | 0.599 (0.533–0.664) |
| Procalcitonin, quartiles | 1.35 | 1.06–1.72 | 0.02 | — |
| Age (years) | 1.02 | 1.01–1.04 | 0.002 | — |
| Pathogen genus, *Streptococcus* | Not retained in the logistic regression model | | | — |
| Pathogen genus, *Staphylococcus* | Not retained in the logistic regression model | | | — |
| Pathogen genus, *Klebsiella* | Not retained in the logistic regression model | | | — |
| C-reactive protein (mg/L) | Not retained in the logistic regression model | | | — |
| Sepsis at admission | Not retained in the logistic regression model | | | — |
| **Model #3: Plasma PCT, 4^th^ vs. 1^st^–3^rd^ quartiles‡**  **(n = 326)** | — | | | 0.597 (0.532–0.662) |
| Procalcitonin, 4^th^ quartile | 1.73 | 1.13–2.66 | 0.01 | — |
| Age (years) | 1.01 | 1.00–1.02 | 0.005 | — |
| Pathogen genus, *Streptococcus* | Not retained in the logistic regression model | | | — |
| Pathogen genus, *Staphylococcus* | Not retained in the logistic regression model | | | — |
| Pathogen genus, *Klebsiella* | Not retained in the logistic regression model | | | — |
| Sepsis at admission | Not retained in the logistic regression model | | | — |
| **Model #4: Plasma PCT, 4^th^ vs. 1^st^ quartile‡**  **(n = 158)** | — | | | 0.622 (0.542–0.701) |
| Procalcitonin, 4^th^ quartile | 3.08 | 1.32–7.22 | 0.01 | — |
| Age (years) | Not retained in the logistic regression model | | | — |
| Pathogen genus, *Streptococcus* | Not retained in the logistic regression model | | | — |
| Pathogen genus, *Staphylococcus* | Not retained in the logistic regression model | | | — |
| Pathogen genus, *Klebsiella* | Not retained in the logistic regression model | | | — |
| C-reactive protein (mg/L) | Not retained in the logistic regression model | | | — |
| Sepsis at admission | Not retained in the logistic regression model | | | — |

Note. AUROC: area under the receiver operating characteristic curve; PCT: procalcitonin.

* Cox proportional-hazards regression using the forced entry method.

† AUROC of the prognostic indices generated by the Cox proportional-hazards regression model to discriminate between positive and negative cases.

‡ Cox proportional-hazards regression using the stepwise method.

§ C-reactive protein was not used in the model due to collinearity.

**Supplemental Table S6. Distribution of procalcitonin values according to procalcitonin quartiles**

| **PCT quartile** | **Q1** | **Q2** | **Q3** | **Q4** |
| --- | --- | --- | --- | --- |
| N | 113 | 114 | 112 | 113 |
| Median (ng/mL) | 0.24 | 1.19 | 5.41 | 35.21 |
| 25 - 75 P (ng/mL) | 0.14–0.36 | 0.90–2.00 | 3.71–7.99 | 18.74–78.25 |
| Minimum (ng/mL) | 0.05 | 0.60 | 2.56 | 11.65 |
| Maximum (ng/mL) | 0.59 | 2.55 | 11.64 | 315.80 |

**Supplemental Table S7.** **Kaplan-Meier analysis reporting the probability of survival without in-hospital mortality in the 452 studied patients according to baseline plasma procalcitonin quartiles.**

|  | **PCT, quartile 1** | | **PCT, quartile 2** | | **PCT, quartile 3** | | **PCT, quartile 4** | |
| --- | --- | --- | --- | --- | --- | --- | --- | --- |
| **Survival time**  **(days)** | **Probability of**  **survival**  **(%)** | **Standard**  **error**  **(%)** | **Probability of**  **survival**  **(%)** | **Standard**  **error**  **(%)** | **Probability of**  **survival**  **(%)** | **Standard**  **error**  **(%)** | **Probability of**  **survival**  **(%)** | **Standard**  **error**  **(%)** |
| 0 | 96.5 | 1.74 | 97.4 | 1.50 | 98.2 | 1.25 | 93.8 | 2.27 |
| 1 | 95.5 | 1.95 | 96.4 | 1.74 | 94.6 | 2.13 | 90.3 | 2.79 |
| 2 | 94.6 | 2.14 | 95.5 | 1.97 | 92.0 | 2.57 | 87.6 | 3.10 |
| 3 | 93.6 | 2.33 | — | — | 90.2 | 2.82 | 85.0 | 3.36 |
| 4 | — | — | — | — | 88.3 | 3.05 | 84.1 | 3.44 |
| 5 | 92.6 | 2.52 | — | — | — | — | 83.2 | 3.52 |
| 6 | — | — | — | — | 86.4 | 3.28 | — | — |
| 7 | — | — | 94.3 | 2.26 | 85.4 | 3.39 | — | — |
| 8 | — | — | 93.1 | 2.53 | 84.3 | 3.51 | 81.1 | 3.73 |
| 9 | — | — | 90.7 | 3.00 | — | — | 80.0 | 3.83 |
| 10 | — | — | 88.1 | 3.44 | — | — | 77.8 | 4.03 |
| 11 | 91.1 | 2.89 | — | — | — | — | — | — |
| 12 | — | — | — | — | 82.8 | 3.75 | — | — |
| 13 | — | — | — | — | 81.2 | 4.00 | — | — |
| 14 | — | — | — | — | 79.6 | 4.24 | 76.2 | 4.25 |
| 15 | 89.0 | 3.49 | 86.3 | 3.81 | — | — | — | — |
| 16 | — | — | — | — | 77.7 | 4.56 | — | — |
| 17 | — | — | — | — | 75.6 | 4.87 | 74.5 | 4.50 |
| 18 | — | — | 84.1 | 4.28 | — | — | 72.7 | 4.73 |
| 19 | — | — | — | — | — | — | — | — |
| 20 | — | — | 79.0 | 5.33 | — | — | — | — |
| 21 | — | — | — | — | — | — | — | — |
| 22 | — | — | 76.3 | 5.80 | — | — | 68.1 | 5.42 |
| 23 | — | — | — | — | — | — | 65.7 | 5.74 |
| 24 | — | — | — | — | — | — | 60.7 | 6.32 |
| 25 | — | — | — | — | — | — | — | — |
| 26 | — | — | — | — | — | — | — | — |
| 27 | — | — | — | — | 71.4 | 6.15 | — | — |
| 28 | 82.2 | 7.33 | — | — | — | — | 56.9 | 6.97 |
| 29 | — | — | 72.1 | 6.85 | — | — | — | — |
| 30 | — | — | — | — | — | — | — | — |
| 31 | — | — | 66.0 | 8.52 | 65.5 | 8.02 | — | — |
| 32 | — | — | 60.0 | 9.63 | — | — | — | — |
| 33 | — | — | — | — | — | — | — | — |
| 35 | — | — | — | — | — | — | 51.7 | 8.03 |

**Comparison of survival curves (Log-rank test)**

| Chi-squared | 11.3473 |
| --- | --- |
| DF | 3 |
| Significance | P = 0.01 |

**Log-rank test for trend**

| Chi-squared (trend) | 11.0784 |
| --- | --- |
| DF | 1 |
| Significance | P = 0.0009 |

**Supplemental Table S8. Hazard ratios and 95% confidence interval for the pairwise comparison of survival probabilities without in-hospital mortality according to baseline procalcitonin quartiles.**

| Factor | **PCT, quartile 1** | **PCT, quartile 2** | **PCT, quartile 3** | **PCT, quartile 4** |
| --- | --- | --- | --- | --- |
| **PCT, quartile 1** | — | 1.62 0.89–2.93 | 2.10 1.15–3.81 | 2.87 1.58–5.19 |
| **PCT, quartile 2** | 0.62 0.34–1.12 | — | 1.30 0.72–2.33 | 1.77 0.99–3.17 |
| **PCT, quartile 3** | 0.48 0.26–0.87 | 0.77 0.43–1.39 | — | 1.37 0.76–2.46 |
| **PCT, quartile 4** | 0.35 0.19–0.63 | 0.56 0.32–1.01 | 0.73 0.41–1.32 | — |

**SUPPLEMENTAL FIGURES**

**Supplemental Figure S1.** Flow diagram of patient selection.


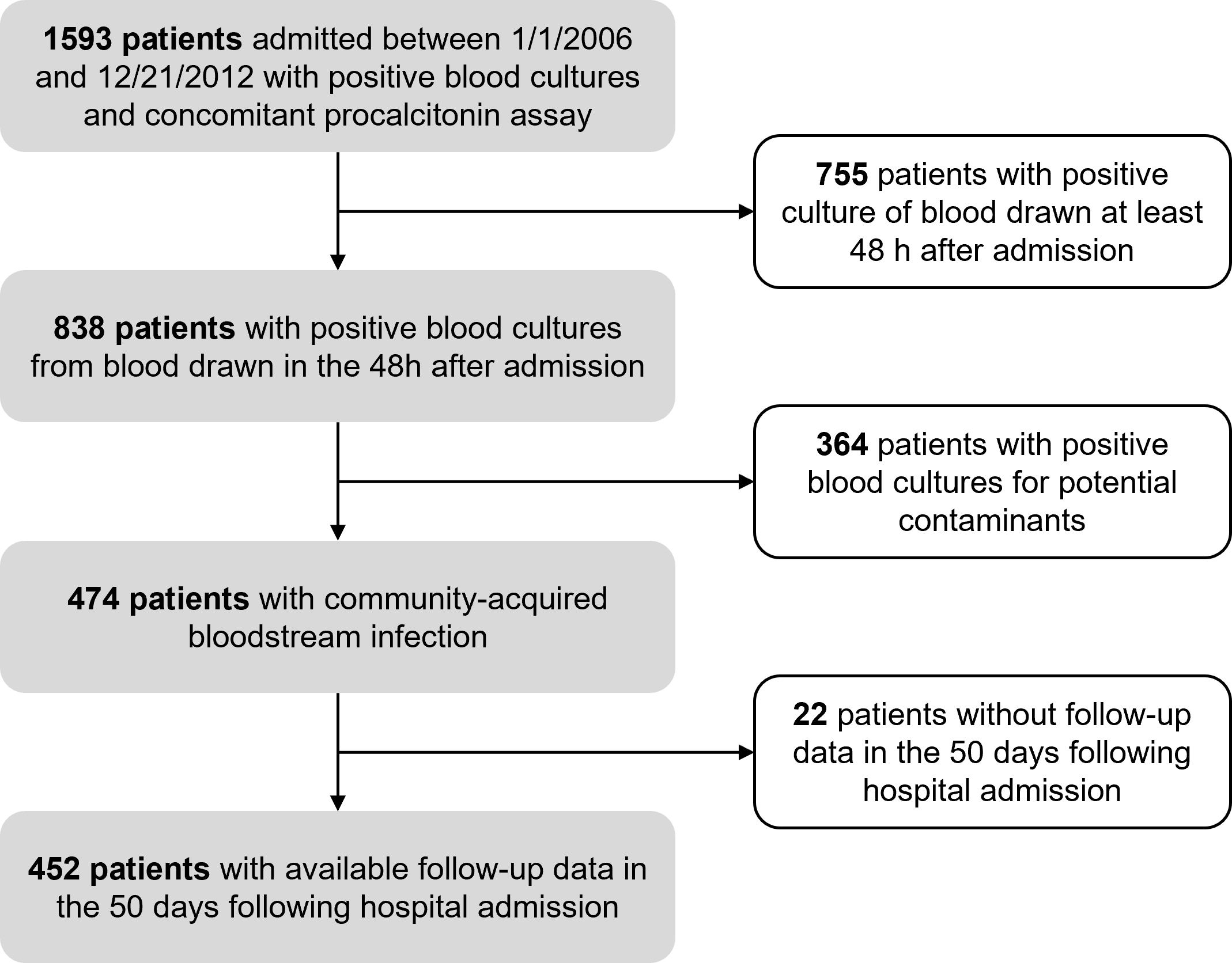


**Supplemental Figure S2. (A)** Kaplan-Meier analysis reporting the probability of survival without in-hospital mortality according to the ROC-defined plasma troponin threshold (> 0.05 ng/mL) at baseline; **(B)** Kaplan-Meier analysis reporting the probability of survival without in-hospital mortality according to the ROC-defined plasma lactates threshold (> 4.24 ng/mL) at baseline.


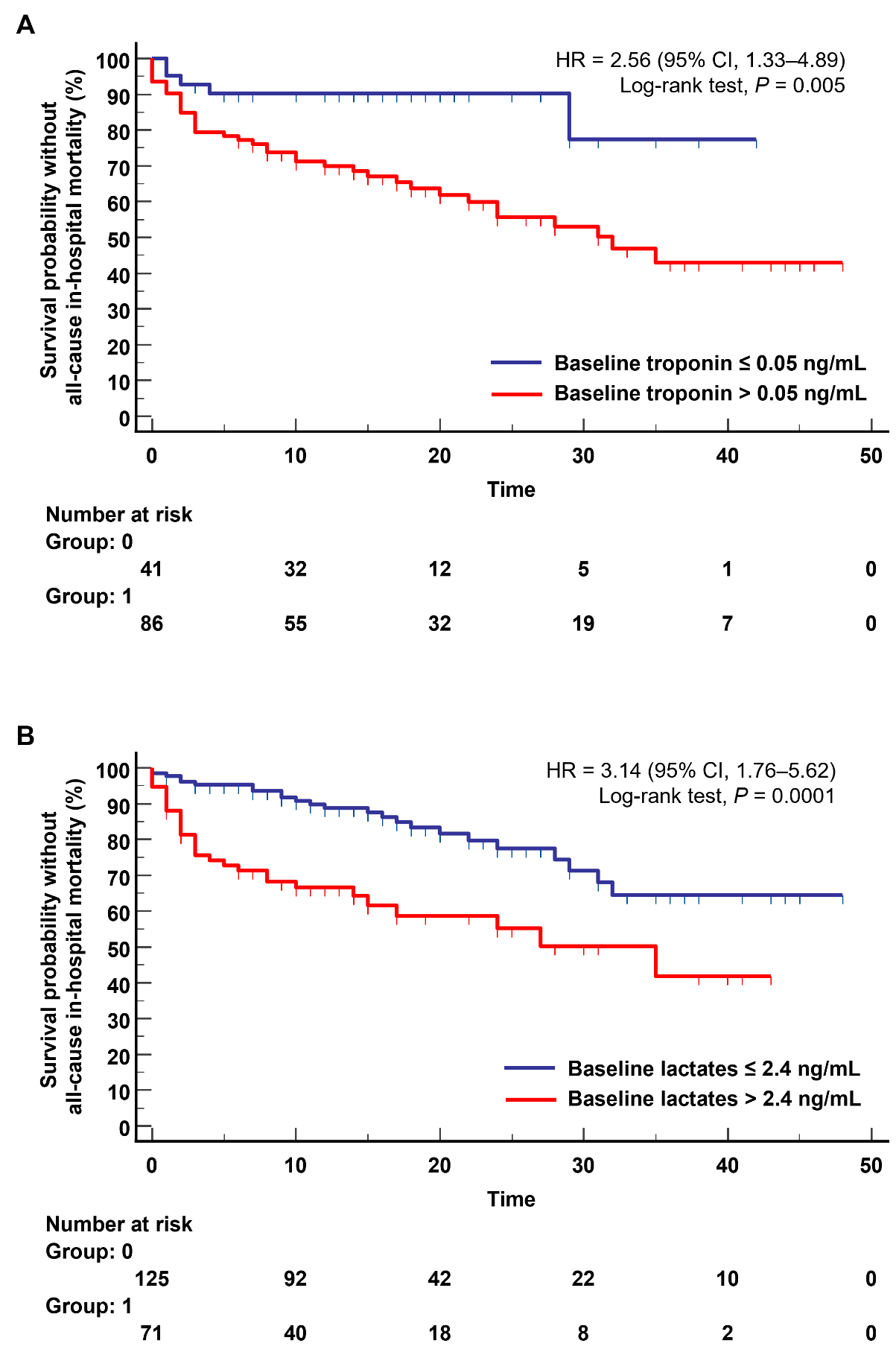


**Supplemental Figure S3.** Probit sigmoid dose-response curve showing the propensity score predicted by the logistic regression model as the dose variable and the risk of 50-day in-hospital mortality as the response variable. The red line shows the probability and corresponding dose. The dashed curves represent the 95% confidence interval for the respective dose. The dose and 95% confidence interval corresponding with a particular probability are taken from a horizontal line at that probability level.


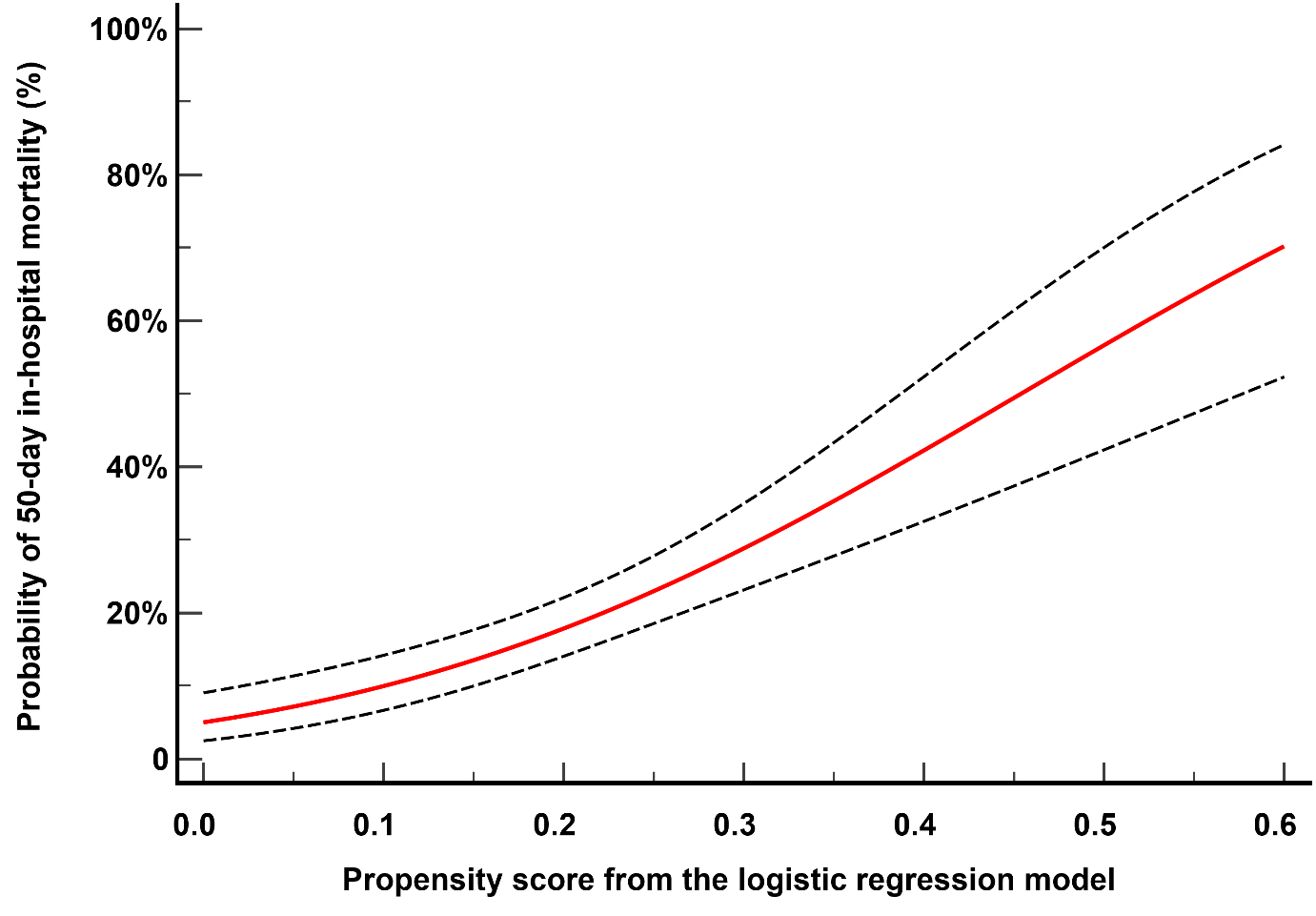


**Supplemental Figure S4. (A)** Kaplan-Meier analysis reporting the probability of survival without in-hospital mortality in the 452 studied patients. The dashed lines represent the 95% confidence interval of the survival probabilities; **(B)** Kaplan-Meier analysis reporting the probability of survival without in-hospital mortality according to the ROC-defined plasma procalcitonin threshold (> 4.24 ng/mL) at baseline.


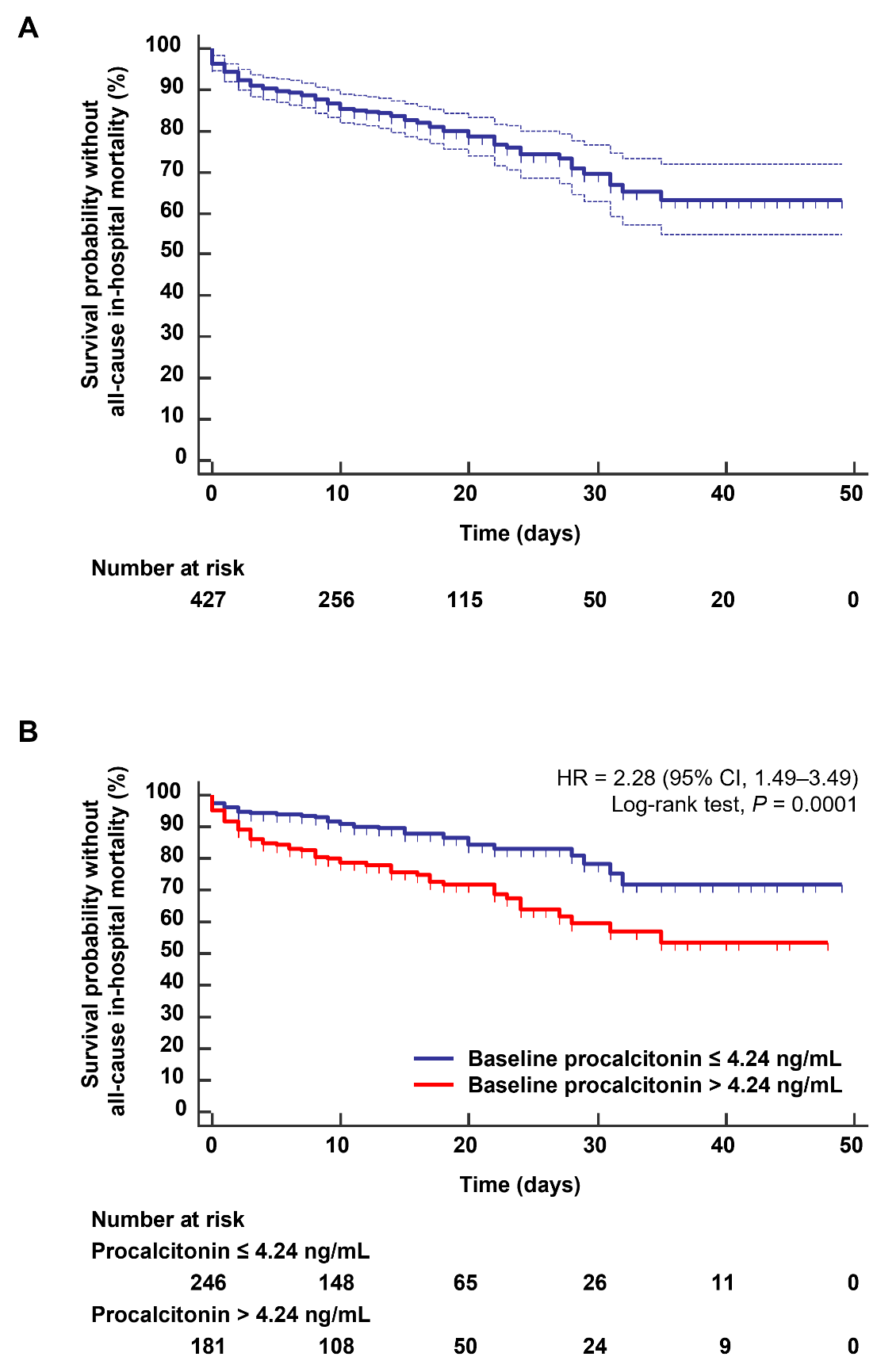

Supplement: Supplementary file 1 — Additional file 1: Supplemental methods. Supplemental Table S1. Probit regression analysis for the propensity score predicted by the logistic regression model as the dose variable and the risk of 50-day in-hospital mortality as the response variable. Supplemental Table 2. Association between plasma procalcitonin level at admission and all-cause 50-day mortality in multivariable logistic regression analysis. Supplemental Table S3. Kaplan-Meier analysis reporting the probability of survival without in-hospital mortality according to the ROC-defined plasma procalcitonin threshold (> 4.24 ng/mL) at baseline. Supplemental Table S4. Hazard ratios and 95% confidence interval for the pairwise comparison of survival probabilities without in-hospital mortality according to the ROC-defined plasma procalcitonin threshold (> 4.24 ng/mL) at baseline. Supplemental Table S5. Association between plasma procalcitonin level at admission and all-cause in-hospital mortality in multivariable Cox proportional-hazards regression. Supplemental Table S6. Distribution of procalcitonin values according to procalcitonin quartiles. Supplemental Table S7. Kaplan-Meier analysis reporting the probability of survival without in-hospital mortality in the 452 studied patients according to baseline plasma procalcitonin quartiles. Supplemental Table S8. Hazard ratios and 95% confidence interval for the pairwise comparison of survival probabilities without in-hospital mortality according to baseline procalcitonin quartiles. Supplemental Figure S1. Flow diagram of patient selection. Supplemental Figure S2. (A) Kaplan-Meier analysis reporting the probability of survival without in-hospital mortality according to the ROC-defined plasma troponin threshold (> 0.05 ng/mL) at baseline; (B) Kaplan-Meier analysis reporting the probability of survival without in-hospital mortality according to the ROC-defined plasma lactates threshold (> 4.24 ng/mL) at baseline. Supplemental Figure S3. Probit sigmoid dose-response [file 40364_2023_450_MOESM1_ESM.docx]
